# The Role of Exosomal miRNAs in Glioma: Biological Function and Clinical Application

**DOI:** 10.3389/fonc.2021.686369

**Published:** 2021-09-01

**Authors:** Yirizhati Aili, Nuersimanguli Maimaitiming, Yusufu Mahemuti, Hu Qin, Yongxin Wang, Zengliang Wang

**Affiliations:** ^1^Department of Neurosurgery, The First Affiliated Hospital of Xinjiang Medical University, Xinjiang, China; ^2^Department of Oncology, The First Affiliated Hospital of Xinjiang Medical University, Xinjiang, China

**Keywords:** glioma, exosomes, miRNA, microenvironment, biomarkers

## Abstract

Gliomas are complex and heterogeneous central nervous system tumors with poor prognosis. Despite the increasing development of aggressive combination therapies, the prognosis of glioma is generally unsatisfactory. Exosomal microRNA (miRNA) has been successfully used in other diseases as a reliable biomarker and even therapeutic target. Recent studies show that exosomal miRNA plays an important role in glioma occurrence, development, invasion, metastasis, and treatment resistance. However, the association of exosomal miRNA between glioma has not been systemically characterized. This will provide a theoretical basis for us to further explore the relationship between exosomal miRNAs and glioma and also has a positive clinical significance in the innovative diagnosis and treatment of glioma.

## Introduction

Glioma is the most common malignant primary tumor of the central nervous system (CNS). Glioblastoma (GBM) has the highest malignant degree, causing thousands of deaths worldwide, with an average median survival time of only 14–16 months (1). Currently, standard GBM treatments include maximal safe surgical resection and combined radio-chemotherapy. Apart from the rapid proliferation, extensive invasion, intra- and intertumoral genetic heterogeneity, and treatment resistance of GBM, the dismal prognosis of GBM patients also originates from poor understanding of molecular pathogenesis, lack of timely diagnosis, and sensitive therapeutic monitoring tools (2). Therefore, it is crucial to elucidate molecular mechanisms underlying glioma development and progression and further explore reliable biomarkers.

In recent years, many researchers have discovered that microvesicles (MVs) including many different types of exosomes can transport various nucleic acids, proteins, and lipids across different tissues from glioma patients (3). Increasing studies have proved that exosomes can regulate the microenvironment of tumors by mediating cell-to-cell communication and play an essential role in tumor occurrence and development, invasion, metastasis, and immune monitoring. Specifically, In the process of tumor deterioration, the release of tumor-cells-derived exosome (T-exo) was significantly increased compared with normal cells, and they carried specific nucleic acid associated with tumor progression (4). Furthermore, through next-generation sequencing (NGS), the expression profiles of various nucleic acids in the T-exo are found, among which the expression profiles of microRNAs (miRNAs) present obvious differential expression. Therefore, T-Exo and its miRNAs have become a research hotspot in the field of cancer in recent years due to their other characteristics and biological functions (5). Similarly, miRNAs regulate glioma cells and tumor microenvironment through exosome and transcriptome pathways, which is expected to provide a new idea for the precise treatment of glioma (6). Therefore, in this review, we summarized the most recent understanding of the T-exo and different exosomal miRNAs on glioma invasion, migration, angiogenesis, and drug resistance, and microenvironment regulation to shed light upon identifying novel therapeutic targets.

## Biogenesis and Characteristics of Exosome

Exosome is a lipid bilayer vesicle with a 40–160-nm diameter, which is cup or saucer shaped under the electron microscope. Unlike other extracellular vesicles (EVs), it is formed by the fusion of small intracellular vesicles before releasing to extracellular spaces (7). Johnstone et al. (8) first found that cells can secrete a membrane body composed of transferrin receptor and membrane-related proteins during reticulocyte maturation in 1983 and then named it “exosome.” Traditionally, this process was only considered as a way for cells to eliminate waste proteins and molecules. However, with the rapid development of exosome extraction and detection techniques, recent studies suggest that mast cells, epithelial cells, neuron cells, and other cells can release exosomes (9), which stably exist in a variety of body fluids, including blood, urine, and cerebrospinal fluid (CSF) (10), and involve in different physiological processes and different disease pathogeneses (11). In addition, exosome is regarded as an essential medium to connect cellular communication; exosomes have been widely studied in the world and favored by more and more scientific researchers.

**Figure 1:**
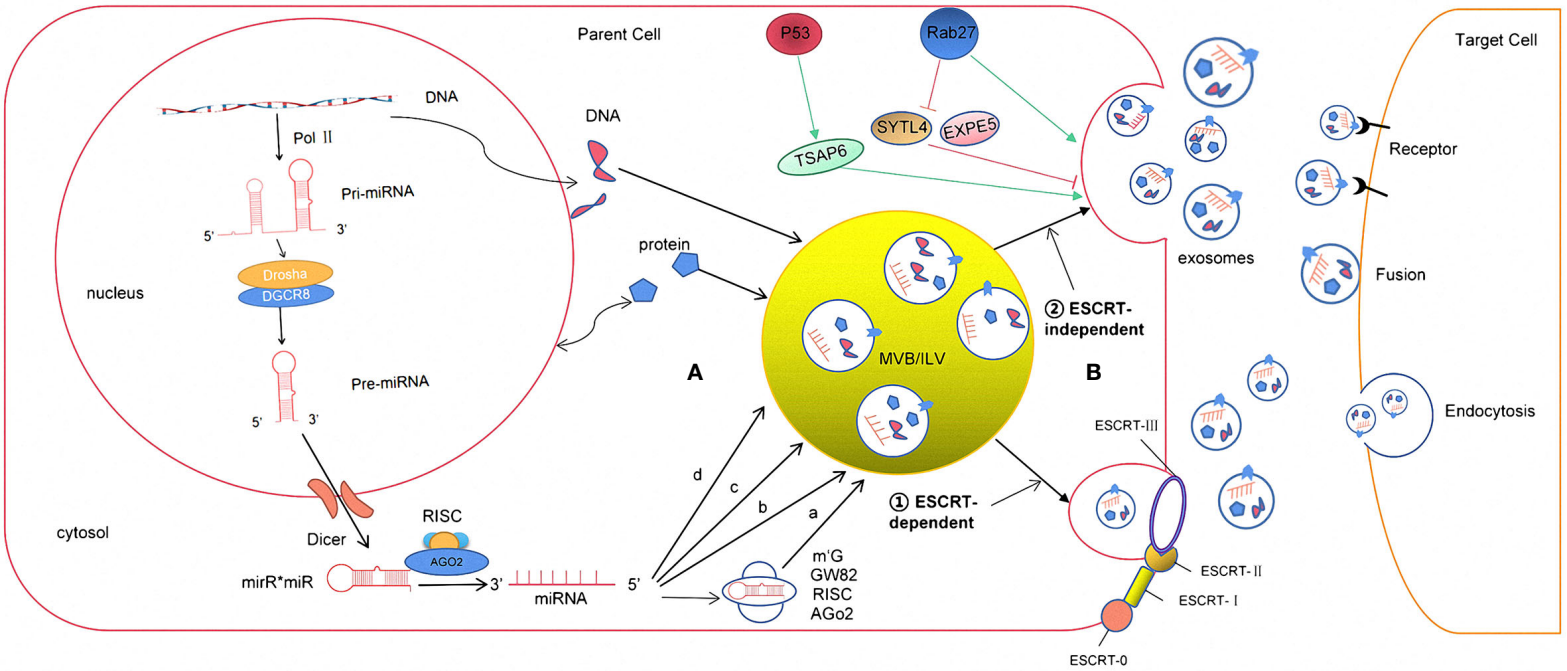
The formation and release process of exosome miRNA were revealed. **(A)** The sorting mechanism of exosomal miRNA MiRNA genes are transcribed into primary miRNAs (pri-miRNA) by Pol-II. Then, with the catalytic action of DGCR8 and Drosha complex, pri-RNA are transmitted into pre-miRNA, which are exported out of the nucleus by exportin5 complex. In the cytoplasm, the pre-miRNAs are digested by the Dicer complex into double-stranded miRNAs, which turn to be single-stranded ones, mature miRNAs, in the next step by Helicase. Mature miRNAs are sorted into exosomes *via* four potential modes: ****(a) neutral sphingomyelinase 2 (nSMase2)-dependent pathway. ****(b) miRNAs motif and heterogeneous ribonucleoprotein (hnRNPs)-dependent pathway; (c) miRNAs sequence 3′-terminal-dependent pathway; (d) Y-box binding protein-1 (YBX-1)-dependent pathway. **(B)** The biogenesis of exosome begins at endosome formation through endocytosis at the plasma membrane, and then, early endosomes maturate to multivesicular bodies (MVB). Exosomes are formed as intraluminal vesicles in MVBs though endosomal sorting complexes required for transport (ESCRT) dependent or independent pathway. Generally, MVBs either fuse with the lysosome for degradation or fuse with the plasma membrane, which results in exosomes secretion. After secretion, exosomes uptake by target cells is mediated by endocytosis, fusion with the plasma membrane, or ligand/receptor interaction.

Exosomes are generated in a process that involves double invagination of the plasma membrane and the formation of intracellular multivesicular bodies (MVBs) containing intraluminal vesicles (ILVs). ILVs are ultimately secreted as exosomes with a size range of ~40–160 nm in diameter through MVB fusion to the plasma membrane and exocytosis. The first invagination of the plasma membrane forms a cup-shaped structure that includes cell-surface proteins and soluble proteins associated with the extracellular milieu. This leads to the *de novo* formation of an early-sorting endosome (ESE) and, in some cases, may directly merge with a preexisting ESE. The trans-Golgi network and endoplasmic reticulum can also contribute to the formation and the content of the ESE. ESEs can mature into late-sorting endosomes (LSEs) and eventually generate MVBs, which are also called multivesicular endosomes (12). MVBs form by inward invagination of the endosomal limiting membrane (that is, double invagination of the plasma membrane). This process results in MVBs containing several ILVs (future exosomes). The MVB can either fuse with lysosomes or autophagosomes to be degraded or fuse with the plasma membrane to release the contained ILVs as exosomes (13). The Ras-related protein GTPase Rab, Sytenin1, TSG101 (tumor susceptibility gene 101), ALIX (apoptosis-linked gene 2-interacting protein X), syndecan-1, ESCRT (endosomal sorting complexes required for transport) proteins, phospholipids, tetraspanins, ceramides, sphingomyelinases, and SNARE [soluble nethylmaleimide-sensitive factor (NSF) attachment protein receptor] complex proteins are involved in the origin and biogenesis process of exosomes, although their precise rate-limiting actions and functions in exosome biogenesis require further in-depth exploration, especially *in vivo* (**Figure 1**).

The effects of exosomes on recipient cells can be different because of their varied expression of cell surface receptors, and such functional heterogeneity can result in one set of exosomes inducing cell survival, another set inducing apoptosis, and a different set inducing immuno-modulation, in different target cell types. Heterogeneity can also be based on the organ and tissue of origin of the exosomes, including whether they are from cancer cells, giving them distinct properties such as tropism to certain organs and uptake by specific cell types (12–14). A combination of all of these features would have the potential to give rise to a higher order of complexity and heterogeneity of exosomes.

In addition, exosomes, as a natural intercellular information carrier with their endogenous and nanoscale volume and their important role in cell communication, have two specific advantages compared to other carrier molecules, especially showing great potential in tumor therapy. The first is its endogenous nature and therefore low immunogenicity (15). Exosomes can reach various tissues and organs through the circulation and release the contents without triggering the immune response (16). Second, a series of changes have taken place in its composition when released by cells during the onset of disease (17), which makes the exosomes membrane surface or vesicles that carry proteins, messenger RNA (mRNA), or miRNA target to recognize the diseased cells and transfer these molecules to the target cells (18). These properties of exosomes provide a broad theoretical basis as drug carriers for biotherapy.

The intensity of the exosomes is rich in a variety of proteins, nucleic acids, lipids, etc. According to the statistics of the ExoCarta database, 3,408 mRNAs, 2,838 miRNAs, 9,769 proteins, and 1,116 kinds of fats have been known to be associated with exosomes. When exosomes receive regulatory signals, autosilent molecules and target cell surface receptor passes combine and act as a control of target cells (19). More importantly, the types and qualities of these contents are closely related to the origin cells. The inherent components also change with the physiological state (20). During the development of glioma, tumor cells can deliver miRNAs to other target cells through the transfer pathways of the exosomes (21) and participate in intercell communication. Later, the containing miRNA binds to the targeting mRNA 3′ noncoding region (UTR) and directly degrades mRNA molecules or prevent mRNA translation (22). At the same time, since miRNA is more stable than mRNA and can be detected in the exosomes, the exosomal miRNA has been identified as a promising biomarker in different diseases (23). In addition, exosomes have a unique nanometer diameter, which makes them freely pass through the blood–brain barrier and blood–cerebrospinal fluid barrier, as a “favorite” field in the research of glioma diagnosis and treatment in recent years (24). Therefore, the role of exosomal miRNA in the occurrence, invasion, immune regulation, and treatment of glioma will be summarized and discussed in this review.

## Exosomal miRNAs

Exosomes contain many kinds of RNA molecules, which are mainly non-coding RNA, such as long non-coding RNAs (lncRNAs), circular RNAs (circRNAs), and miRNAs, among which miRNAs have the highest content and play an important role in the regulation of gene expression, so they have received the most attention (25). The ExoCarta database shows that 2,838 miRNAs have been found in exosomes, of which about 26 are closely related to gliomas (26). miRNAs are a group of non-coding small RNAs about 22–26nt in length. They can stably exist in different body fluids because they can enter the exosome, and some of them can even be loaded on high-density lipoprotein, which protects them from being degraded by RNA enzymes (27). As to the studies of miRNA expression, Goldie et al. (28) found that the content of miRNAs in exosomes was significantly higher than that in its source cells. Guduric et al. (29). found a similar phenomenon through comparative analysis of the expression level of miRNAs in many cell lines and their corresponding derived exosomes. It was found that miRNAs (such as miR-150, miR-142, and miR-451) could enter the exosome before other RNA molecules and exosomes had an enrichment effect on miRNAs, which might explain the increasing expression level compared to its source cells. However, the mechanism has not been clarified in this study (30). In addition, other studies have shown that the expression levels of exosomal miRNAs secreted by cells depend on the physiological/pathological conditions. Compared to healthy individuals, the expression level of plasma exosomal miR-21 was significantly increased in patients with malignant gliomas (31). Thuringer (32) and other studies (33) indicated that exosomal miR-5096 obtained from patients with GBM promoted the growth of filopodium and the invasion of glioma cells by regulating inward rectifier K^+^ channel Kir4.1. At the same time, it also increased the release of exosomes, thus further promoted the invasion and migration of GBM. The above studies suggest that the loading of the miRNA into exosomes are specific to its sequences, enzymes, or related proteins. However, the specific loading mechanism is not completely clear (34). According to the conclusions obtained so far (35), it may be associated with the following pathways: (1) neutral sphingomyelinase 2 (nSMase2)-dependent pathway; (2) miRNAs motif and heterogeneous ribonucleoprotein (hnRNPs)-dependent pathway; (3) miRNAs sequence 3′-terminal-dependent pathway; and (4) Y-box binding protein-1 (YBX-1)-dependent pathway (**Figure 1**).

Extensive studies suggested that miRNAs are selectively sorted into exosomes and participate in cell-to-cell communication in tumor microenvironment and play an essential part in cancer biology. Furthermore, the easy access, abundance, and stability of exosomal miRNAs in biofluids have made them ideal biomarkers for various cancers, including gliomas.

## The Functional Role of Exosomal miRNAs in Glioma

**Figure 2:**
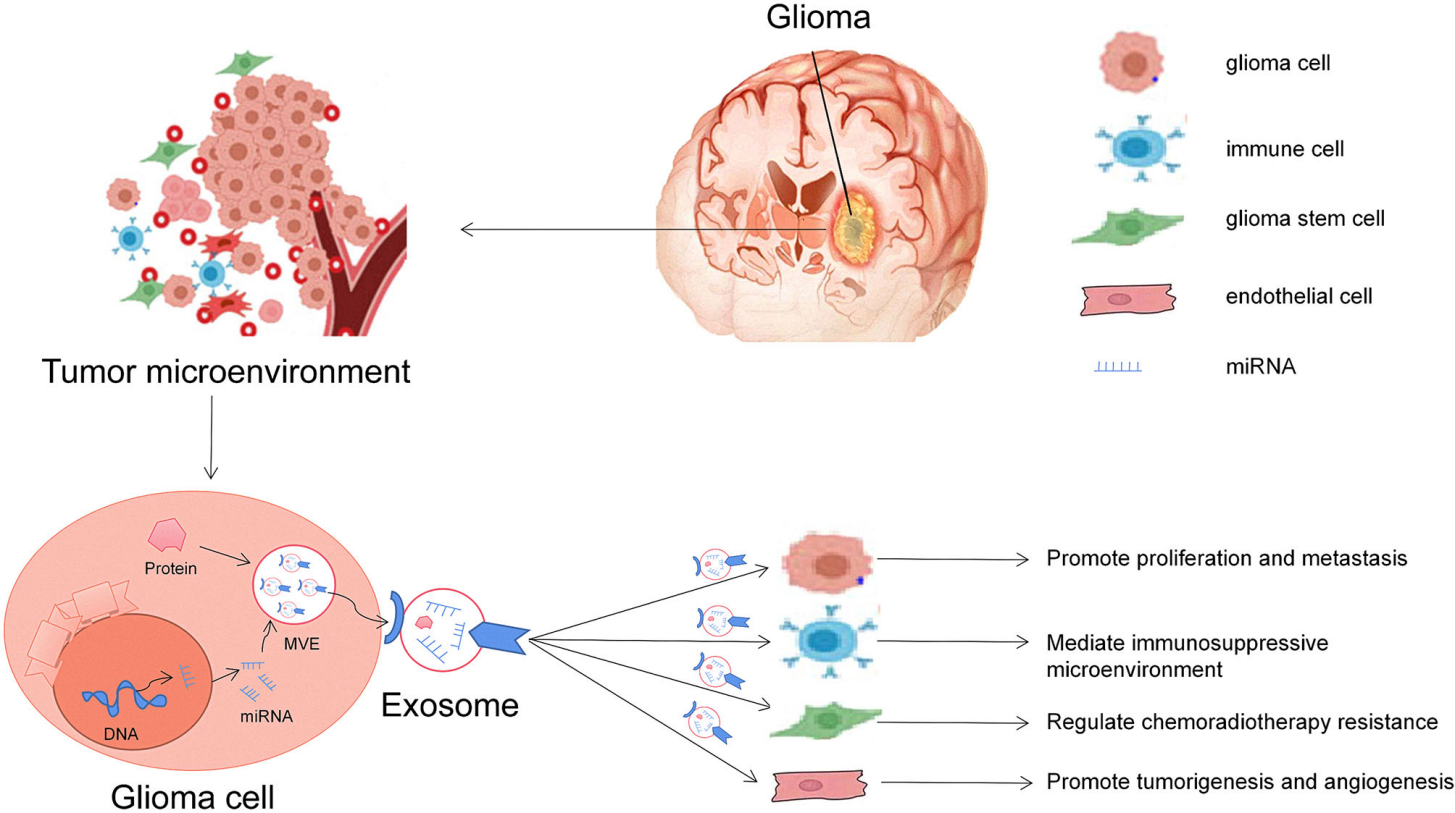
Roles of glioma-derived exosomes in glioma development. The first general mechanism is glioma cells can communicate with other cells *via* exosomal miRNAs in the tumor microenvironment. The second general mechanism is that exosomes derived from glioma cells alter the behavior of normal cells, including promoting glioma carcinogenesis, angiogenesis, and drug resistance and helping cancer cell escape from host immune system, and can be useful diagnostic and/or prognostic biomarkers.

Glioma is a complex and heterogeneous tumor. It not only contains tumor cells but also has many kinds of non-tumoral cell types, such as astrocytes, microglia, endothelial cells, and immune cells, which together constitute the complex glioma microenvironment (13). miRNAs are one of the essential gene regulatory factors in the body. It has been found that a single miRNA can regulate multiple targeted mRNAs (36). Similarly, a single targeted mRNA can also be regulated by multiple miRNAs. Through this regulatory mechanism, when tumors occur, exosomes can release selectively enriched miRNAs to activate specific signal pathways in the target cells and affect the microenvironment of gliomas (37). Therefore, exosomal miRNAs play an essential role in the proliferation, metastasis, drug resistance, and immunity of gliomas (**Figure 2**).

### Exosomal miRNAs and Cell Proliferation/Invasion in Glioma

Many researchers have found that gliomas release exosomes during disease progression. These exosomes can deliver miRNAs to surrounding normal cells through endocytosis or lipid membrane fusion, thereby destroying the homeostasis of normal cells. It shows tumor characteristics in biological behavior and promotes malignant cell proliferation and invasion (38). Compared with normal brain-tissue-derived exosomes, the expressions of miR-222, miR-9, and miR-26a in glioma-derived exosomes were significantly increased, and many signal transduction pathways were activated to stimulate tumor growth (39). It has been reported that the secretion of miR-1246 from exosomes, which binds to the mRNA of cell adhesion molecule-1 (CADM1) gene, was increased in malignant GBM and promoted the proliferation and migration of glioma cells (40).

Figuroa et al. (41) found a new GBM matrix component human gliomas as glioma-associated human mesenchymal stem cells (GA-hMSCs). miR-1587 is highly enriched in its derived exosomes, which can promote the proliferation of glioma stem-like cells and enhance their tumorigenicity. On the contrary, miRNAs, which inhibit the growth of glioma, also exist in the glioma-derived exosomes. Studies have shown that Sakr et al. (42) transfection of miR-150-5p or miR-133a mimics into the exosomes from glioma cells and cocultured the exosomes with glioma cells. Those exosomes inhibited membrane type 1 matrix metalloproteinases (MT1-MMP) expression and then induced apoptosis of glioma cells. However, this study has not been verified *in vitro* experiments and is still lacking in controversy. Lei et al. (43) were the first to identify that overexpression of miR-199a in GA-hMSCs exosomes inhibited the glioma growth by reducing the expression of Arf GTP enzyme activating protein 2 (AGAP2). The above studies show that exosomal miRNAs are closely related to the proliferation and apoptosis of gliomas, which provides a new perspective for understanding the mechanism of the occurrence and development of gliomas and provides a potential novel target for gene therapy.

The invasion and metastasis of glioma is a complex process facilitated by many factors. Both tumor cells and surrounding stromal cells can secrete exosomes to change the microenvironment during metastasis through the miRNAs (43). For example, the level of circulating exosomal miR-148a in serum of patients with GBM significantly increased and enhanced the proliferation of glioma cell proliferation and facilitated the metastasis by activating the STAT3 signal pathway to inhibit expression of target gene CADM1 (44). Similarly, Thuringer (32) and other researchers (33) found that glioblastoma-derived exosomal miR-5096 continuously stimulated tumor cells to secrete more exosomes containing miR-5096 and increased the number of motor filamentous pseudopodia. In addition, the study also proved the association between the K^+^ channel and GBM. Through follow-up experiments, exosomal miR-5096 could transfer from GBM cells to astrocytes through heterotypic gap junction and inhibit inward rectifier potassium channel (Kir4.1) protein in GBM cells and then improve their invasive capacity (45) (**Table 1**). Many studies further confirmed the promoting effect of glioma-derived exosomal miRNA on the growth and invasion of glioma cells through *in vivo* and *in vitro* experiments.

**Table 1:** The biological functions of exosomal miRNAs in glioma.

miRNA	Parent cell	Target cell	Pathway/target(s)	Biological function	Reference
miR-301a	Hypoxic-glioma cells	Normoxia glioma cells	Wnt/s-catenin	Promote radiation resistance	(46)
miR-151a	TMZ-resistant GBM cells	TMZ-sensitive GBM cells	Inhibiting XRCC4-mediated DNA repair	Enhances chemosensitivity to TMZ	(47)
miR-21	glioma cells	Microglia	*Bmpr2/Btg2*	Mediate immunosuppressive microenvironment	(48)
miR-1246	Hypoxic-glioma cells	Macrophages	NF-κB	Mediate immunosuppressive microenvironment	(49)
miR-29a, miR-92a	Hypoxic-glioma cells	Myeloid-derived suppressor cells	microRNA-29a/Hbp1/microRNA-92a/Prkar1a	Mediate immunosuppressive microenvironment	(50)
miR-10a, miR-21	Hypoxic-glioma cells	Myeloid-derived suppressor cells	miR-10a/Rora and miR-21/PTEN	Mediate immunosuppressive microenvironment	(51)
miR-9	glioma cells	Endothelial cells	MYC and OCT4	Promote tumorigenesis and angiogenesis	(39)
miR-21	Glioma steam cells	Endothelial cells	miR-21/VEGF	Promotes angiogenesis	(52)
miR-26a	Glioma steam cells	Endothelial cells	Activated the PI3K-Akt	Promotes angiogenesis	(53)
miR-375	Marrow stromal cells	Glioma cells	Inhibiting SLC31A1	Inhibit glioma progression	(54)
miR-199a	Mesenchymal stem cells	Glioma cells	Downregulating AGAP2.	Inhibit proliferation, invasion, and enhance chemosensitivity	(43)
miR-124a	Mesenchymal stem cells	Glioma steam cells	Silencing forkhead box (FOX)A2	Antiglioma agent	(55)
miR-146b	Marrow stromal cells	Gliosarcoma cells	EGFR/NF-kB	Reduce glioma growth *in vivo*	(56)
miR-584	Mesenchymal stem cells	Glioma cells	AKT and MAPK	Suppress tumor progress	(57)
miR-7	Mesenchymal stem cells	Glioblastoma cells	miR-7-XIAP	Increase apoptosis and suppress growth	(58)
miR-302-367	Glioma steam cells	Glioblastoma cells	CXCR4/SDF1	Inhibit growth	(59)
miR-124	Mesenchymal stem cells	Glioblastoma cells	Inhibiting CDK6	Inhibit proliferation, migration, and confer chemosensitivity	(60)
miR-1	Glioblastoma cells	Endothelial cells and glioblastoma cells	ANXA2	Inhibit angiogenesis, invasion, and neurosphere formation	(61)
miR-1587	Mesenchymal stem cells	Glioma stem cells	Inhibiting NCOR1	Increase tumorigenicity	(41)
miR-451/21	Glioma cells	Microglia/macrophages	kinase B1 (LKB1)/AMP-activated protein kinase	Promote proliferation and immune suppression	(62)
miR-148a	Glioma cells	Glioma cells	CADM1/STAT3	Promote proliferation and metastasis	(44)
miR-221	U87MG	SHG-44	DNM3	Promote proliferation, migration, and TMZ resistance	(63)
miR-1238	Glioblastoma cells	Temozolomide-sensitive cells	EGFR-PI3K-Akt-mTOR	Promote proliferation, migration, and TMZ resistance	(30)

On the contrary, Sharif et al. (60) extracted umbilical cord Wharton’s jelly-derived mesenchymal stromal cells (WJ-MSCs) released exosomes that could deliver miR-124 to GBM cells and impaired their resistance to temozolomide (TMZ) chemotherapeutic drugs and their invasion and migration capacities. At the same time, they also demonstrated in luciferase reporting experiments that this biological process required the participation of the target gene cyclin-dependent kinase 6 (CDK6) (64). In summary, the high invasiveness and metastatic ability of tumor cells are essential markers for malignant tumors. The rapid development of molecular biology technology has removed many obstacles in discovering the critical molecules of tumor progression. By accurately screening differentially expressed exosomal miRNAs or their related proteins, further exploration of their function and mechanism will help us to better understand the pathogenesis of GBM. At the same time, more studies on the mechanism of exosomal RNA in glioma growth process are needed to further search for reliable specific biomarkers.

### Exosomal miRNAs and Angiogenesis/Tumor Metabolism in Glioma

Glioma is one of the tumors with the highest blood vessel density (65). Giusti et al. (66) found that exosomal miRNAs derived from glioma cells can directly or indirectly affect the proliferation, migration, differentiation, and formation of new tubular structures of vascular endothelial cells. Sun (52) and other studies (67) have shown that the exosomes secreted by glioma stem cells can promote endothelial cell proliferation. The reason is that miR-21 in exosomes activates the VEGF signal pathway and contributes to new tumor angiogenesis. On the contrary, as a tumor suppressor, miR-1 has an entirely different effect on glioma angiogenesis (61, 68). Bronisz et al. (69) have found that glioma-derived exosomes can significantly promote the formation of longer and more branched lumens of human cerebral vascular endothelial cells (HBMECs). The overexpression of MiR-1 in exosomes can effectively inhibit the production of related angiogenic factors (such as VEGF), resulting in a significant decreased angiogenic effect (70). Although the role of exosomal miR-1 in inhibiting glioma angiogenesis has been confirmed, its mechanism and causes are still unclear, which need to be explored in a large number of studies.

There are many metabolic regulation mechanisms in the tumor microenvironment. It has been well studied that the alteration of glucose metabolism is highly associated with the occurrence and development of tumors. During the growth of tumor cells, they can adapt themselves to the tumor microenvironment with low glucose/low ATP by changing their metabolic patterns (71). In a high-glucose microenvironment, the exosome secreted by glioma cells is rich in miR-451 and can regulate glioma’s metabolism to affect the growth, migration, and invasion of tumor cells (62). Ansari et al. (72) indicated that miR-451 directly inhibited the expression of calcium-binding protein 39 (CAB39), which resulted in a decrease in LKB1 activity and eventually inhibited the AMP-activated protein kinase (AMPK) signaling pathway. In the low-glucose microenvironment, the expression of miR-451 is downregulated, and the AMPK signal pathway is activated, which makes glioma cells more adaptable to the low-glucose microenvironment and promotes the disease progression. The study and analysis of the overall metabolism of glioma shows that the metabolic remodeling of glioma cells can affect the progression of glioma (73) (**Table 1**). In summary, glioma-derived exosomal miRNAs regulate tumor microenvironment metabolism and play an essential role in tumor immunoregulation and treatment.

### Exosomal miRNAs as a Double-Action Sword in Glioma

As a means of communication between cells, exosomal miRNAs serve as a double-action sword in glioma. The specific role of exosomes depend on the biological function of the sources and the contents. First of all, many studies have shown that miRNAs carried by tumor exosomes have carcinogenic effects on gliomas, thus promoting tumor growth, invasion, and recurrence (74). Specifically, Karsy (75) and other studies have found that some miRNAs (including miR-21, miR-10b, and miR-26a) in glioma-derived exosomes were upregulated in glial tissue compared with normal brain tissue and can promote the occurrence and development of glioma. Among them, miR-21 can inhibit the expression of programmed cell death protein 422, which promotes apoptosis and reduces the production of reversion-inducing cysteine-rich protein with Kazal motifs (RECK) protein and tissue Inhibitor of metalloproteinase (TIMP), therefore inhibiting matrix metalloproteinases and promoting proliferation and migration of glioma cells (76). Others have found that miR-10b could directly promote the growth of glioma cells by targeting inhibition of proapoptotic protein Bim and cell cycle inhibitors p16 and p21 (77). In addition, Kim (53) discovered that miR-26a can promote the proliferation of glioma cells by inhibiting the production of PTEN, RB1, and MAPK3K2/MEKK2 proteins and activating the AKT signal pathway. The above studies have confirmed the carcinogenic role of various exosomal miRNAs in the development of glioma, providing a possible target for the molecular biology of glioma.

In the meantime, recent studies have found that exosomal miRNAs not only have carcinogenic effects but also have anticancer effects. miR-34, miR-128, and miR-1 in exosomes derived from glioma can inhibit the growth of glioma cells. miR-34 can inhibit the growth of glioma cells by targeting the expression of oncogenes such as Notch-1, Notch-2, c-Met, and platelet-derived growth factor receptor alpha (PDGFRA) (78). Godleweski et al. (79) have shown that miR-128 can simultaneously target two components of PRC-PCR1 (by targeting Bmi1) and PCR2 (by targeting SUZ12) to inhibit the self-renewal and proliferation of glioma stem cells. Peruzzi et al. (80) have shown that the loss of miR-128 expression in early mouse brain cells might be associated with the initiation of the pathogenesis process of glioma. The expression of miR-1 in glioma cells was significantly decreased compared to normal brain tissues. miR-1 could decrease the expression of EGFR, PCR1, PCR2, and p-JNK by targeting ANXA2 and Met, thus inhibiting the growth of glioma cells and tumor angiogenesis and reducing the invasiveness of glioma cells (81). These studies have supplemented the traditional understanding of exosomal miRNA and further explored exosomal miRNAs that have anticancer effects, thus better exploring the potential biological functions of miRNAs in exosomes of tumor cells.

### Exosomal miRNAs and Immunosuppressive Microenvironment

Gliomas affect the surrounding tissues and transform the tumor microenvironment through exosomes to create suitable conditions for tumor development and metastasis. More and more evidence suggest the importance of exosomes and their contents in the communication between tumor cells and other cells (37). Exosome-mediated cell-to-cell communication has been shown to facilitate cell transformation, phenotypic recombination, and functional changes in recipient cells through local and systemic modifications of the microenvironment and direct transfer of biomolecules such as miRNAs. In the glioma microenvironment, glioma stem cells induce bone-marrow-derived monocytes and microglia to differentiate into tumor-associated macrophages (TAMs) by releasing exosomes. A variety of evidence show that TAMs promote tumor recurrence, growth, invasion, and metastasis by affecting tumor metabolism. In addition, the TAMs exosome carrier miR-1246 under an anoxic environment regulates the polarization of M2 macrophage through activating the STAT3 signal pathway while inhibiting the nuclear factor kappa B (NF-κB) signal pathway (49). At the same time, TAMs exosome promotes the release of angiogenic factors from tumor cells and interstitial tumor cells, which eventually promote neovascularization and the formation of the immunosuppressive microenvironment (82). Therefore, immunotherapy in the tumor microenvironment has gradually become the focus of glioma research in recent years.

Increasing studies have proved the importance of exosomal miRNAs in the immune system. During the tumorigenesis, tumor cells release exosomes that carry tumor-specific miRNAs to regulate immune cells, thereby weakening the immune function of host cells and contributing to the glioma cell genesis, immunosuppression, and immune escape. Studies have reported that tumor-derived exosomes can deliver highly enriched miR-203 to immune cells, which eventually promote a variety of immune cell apoptosis, followed by immune escape and rapid tumor growth. As it is all known, myelogenous suppressor cells (MDSCs) play a pivotal role in forming the immunosuppressive microenvironment and tumor immune escape (83). Guo et al. (50) found that tumor exosomal miR-29a and miR-92a stimulated the differentiation of functional MDSCs. In this process, miR-29a and miR-92a silenced HMG box transcription factor 1 (Hbp1) and cAMP-dependent protein kinase regulatory subunit 1α (Prkar1a), respectively, and activated the expansion and activation of myelogenous suppressor cells, thereby enhancing immunosuppression. Guo et al. (50) also proved that glioma-derived exosomal miRNAs were involved in the differentiation of myelogenous suppressor cells and explored the mechanism of tumor immunosuppressive environment *in vitro* and *in vivo*. In *in vitro* cell experiment, they first analyzed the exosomes secreted by GBM in the hypoxic microenvironment and discovered that miR-10a and miR-21 might mediate the amplification and activation of MDSCs through Rora/IκBα/NF-κB and inhibit host cellular immune response through PTEN/PI3K/AKT signaling pathways. In addition, *in vivo* study suggested that the number of MDSCs was significantly reduced in the spleen of glioma mice after transfection of miR-10a or miR-21 (p < 0.05) (51). In another study, Qian et al. (84) found that miR-1246 in the exosomes of glioma cells in hypoxia bound to the 3′ end of the mRNA of human telomere repeat binding factor 2 interacting protein (TERF2IP) gene and inhibited its expression though activating STAT3 signal pathway and inhibiting NF-κB signal pathway, which eventually induced M2 macrophage polarization and promoted the formation of the immunosuppressive tumor microenvironment (**Table 1**). In summary, these studies provided new insights into the role of exosomal miRNAs in immune escape and immunosuppression of glioma cells.

### Exosomal miRNA and Autophagy in Glioma

Autophagy is a widely existed degradation mechanism in cells, which can remove damaged proteins and organelles through the lysosomal pathway and maintain cell homeostasis. While exosomes were initially thought to be a way of shedding reticulocyte receptors, macrophage was initially thought to be merely a cellular waste removal program (85). In recent years, studies have found that exosomes are important transmitters of information between cells, and their potential as biomarkers of disease has attracted more and more attention, while macrophage also plays an important role in everything from irregular secretion to stress adaptation and intercellular communication. It is suggested that there are common molecular and regulatory mechanisms between exosome biogenesis and macroautophagy, and there is a close relationship between them. Exosomes can participate in the occurrence and development of tumor, myocardial ischemia–reperfusion injury, diabetic nephropathy, osteoarthritis, and other diseases by regulating autophagy. The relationship between exosomes and autophagy have become a research hotspot (86). A recent study reported that many exosomal lncRNAs could regulate tumor progression through “lncRNA–miRNA–mRNA” mode. As a dominant regulator of glioma cell autophagy, LINC00470 promoted the expression of ELFN2 through sponge of exosomal miR-101 to distract glioma cell autophagy (87). Activation of autophagy might be a clinical objective that contributes to therapeutic efficiency of immunogenic chemotherapy and/or radiation therapy.

Studies have shown that miRNAs contained in exosomes and autophagy play a regulatory role in the growth of glioma, such as miR-7, miR-181-5p, miR-138, miR-30d-5p, and miR-221/222. Cai et al. (88) found in the study of radiation-induced bystander effect that X-ray-irradiated astrocytes could extract exosomes containing a large number of miR-7, and these exosomes rich in miR-7 promoted the autophagy process of glioma cells by targeting the expression of antiapoptotic protein Bcl-2. However, the pathway was not clearly confirmed in this study, and the molecular mechanism between exosome miR-7 and autophagy still needs to be further explored through *in vivo* and *in vitro* experiments. At the same time, Song et al. (89) found that miR-7 in exosomes can also inhibit the occurrence of autophagy induced by Akt/mTOR signaling pathway by regulating the expression of EGFR, thus promoting the necrosis or apoptosis of glioma cells. In addition, Stojcheva et al. (90) also proved that the apoptosis inhibitor BIM inhibits the enrichment of miR-138 by targeting astrocytes, reduces the concentration of miR-138 in exosomes, and enhances the autophagy reaction of glioma cells, thus inhibiting the drug resistance of glioma cells to TMZ. Although this study was intended to be conducted *in vitro* and the results are controversial, it provides a promising approach to the treatment of glioma.

Exosomes outside the biological occurrence and autophagy in maintaining steady and mitigate cells have a vital role, but a growing body of evidence indicates that exosomes can stimulate tumor cells by regulating tumor microenvironment of autophagy process; tumor cells at the same time also release the secretion in the body through specific microRNAs. TMZ induced the formation of drug resistance. This allows for dynamic cell-to-cell communication. At the molecular level, autophagy-related proteins and protein complexes play a certain role in exosome biogenesis. At the organelle level, exosomes and autophagy pathways intersect at autophagy intosomes, whose inclusions have multiple “fates,” including extracellular release or lysosomal degradation. The dynamic and environmental dependence of the interaction between exosomal miRNAs and autophagy is of great importance to both normal physiology and pathology. Therefore, a clearer understanding of its regulatory mechanism and complexity can provide theoretical support for the clinical treatment of glioma.

### Exosomal miRNA and Chemoradiotherapy Resistance of Glioma

Temozolomide is the first-line chemotherapeutic drug for glioma but sometimes results in resistance. Studies have shown that exosomes could regulate a variety of complex signal pathways by mediating small molecules such as miRNAs, which leads to chemotherapy resistance. Yang et al. (63) suggested that the apoptosis rate of different groups of cells that were treated with temozolomide for 48 h was different, and cells coincubated with exosomes were associated with the lowest apoptosis rate, suggesting that exosomes derived from glioma cells may enhance the chemotherapeutic resistance of temozolomide. Similarly, Yin et al. (91) found that the expression of circulating exosomal miR-1238 in plasma of tumor patients was significantly higher than that of healthy controls. Further studies have shown that tumor exosomal miR-1238 can specifically induce the production of miR-1238 in temozolomide-sensitive cells, which leads to a significant decrease in the expression of target gene cellar protein (CAV1) in receptor cells. The activation of EGFR–PI3K–Akt–mTOR pathway leads to resistance to temozolomide-sensitive cells (30). In addition, miR-151a deletion can also lead to TMZ resistance in GBM cells. Similarly, TMZ-resistant GBM cells can transform TMZ-sensitive GBM cells to TMZ-resistant GBM cells through the DNM3 pathway by releasing exosomes containing a large number of miR-151a and miR-221 (47). The results further confirmed the conclusions of Yang et al. (63) (**Table 1**).

There are similar studies in radiotherapy. Yue et al. (46) found that glioblastoma-derived exosomal miR-301a could specifically inhibit the expression of TCEAL7 in recipient cells in the hypoxic tumor microenvironment by blocking the transport of β-catenin from cytoplasm to nucleus and inhibiting Wnt/β-catenin signal pathway.

In summary, new studies have revealed that exosome-derived miRNAs may be involved in the molecular mechanism of glioma radiotherapy and chemotherapy resistance. With the rapid development of gene chips and the next-generation sequencing technology, critical exosomal miRNAs and associated mechanisms might be identified in the future and novel therapeutic targets as well.

## Clinical Applications of Exosomal miRNAs in Glioma

Most glioma patients are in the middle or late stage when they come to the hospital, and there is a lack of timely and effective treatment. Although the treatment is also improving, the prognosis is still depressing. Finding highly sensitive and specific biomarkers for early monitoring of glioma lesions is still a critical scientific problem to be solved in glioma clinics. In recent years, many studies by researchers have proved that the body fluids of tumor patients have more circulating exosomes than that of healthy humans. Body fluids in these exosomes show significant differences in miRNAs expression, which are similar to the tumor tissues from which they originated (92). Therefore, exosome-mediated miRNAs have potential value as a biomarker for the diagnosis and prognosis of gliomas and can even be developed as a new target for gene therapy, thus opening a new chapter for innovative treatment of gliomas (**Table 2**).

**Table 2:** Clinical application of exosomal miRNA in glioma.

miRNA	Expression	Source	Diagnosis	Prognosis	Response to treatment	Reference
miR-21	Upregulation	CSF/Plasma	Yes	Yes	Yes	(48)
miR-301a	Upregulation	Serum	Yes	Yes		(93)
miR151a	Upregulation	CSF/serum	Yes	Yes		(47)
miR-34s	Upregulation	Serum	Yes	Yes		(94)
miR-218	Upregulation	CSF	Yes			(95)
miR-454-3p	Downregulation	Plasma	Yes		Yes	(96)
miR-210	Upregulation	Plasma	Yes	Yes		(97)
miR-193b	Upregulation	CSF	Yes			(98)
miR-222	Upregulation	Plasma		Yes		(99)
miR-124-3p	Upregulation	Plasma	Yes	Yes		(99)
miR-320	Upregulation	Serum	Yes			(93)
miR-574-3p	Upregulation	Plasma		Yes		(100)
miR-451	Upregulation	Plasma	Yes	Yes		(72)
miR-1238	Upregulation	Plasma			Yes	(91)
miR-221	Upregulation	Plasma			Yes	(93)
miR-10b	Downregulation	CSF		Yes		(101)
miR-148	Upregulation	Serum	Yes			(44)

### Diagnosis

One of the critical reasons for the high mortality rate of glioma is the lack of specific early diagnosis tools, making patients lack effective early treatment. At present, the diagnosis of glioma reply heavily on MRI and histopathological biopsy. However, the specificity of MRI is not high, and it is difficult to identify early lesions smaller than 2.0–3.0 mm (102). As a traumatic examination, pathological biopsy can affect the function of normal brain tissue while not reflecting the dynamic changes in the diseased tissues. The latest study found that glioma-derived exosomal miRNAs stably existed in body fluid. Liquid biopsy has the advantages of accuracy, high efficiency, and minimally invasive, which can make up for the deficiency of traditional examination (103). However, the value of liquid biopsy and circulating exosomal miRNAs in patients with gliomas has been controversial. Therefore, many studies have explored and confirmed the significance of circulating exosomal miRNA in the early diagnosis of gliomas. Manterola et al. (100) used gene chip technology to detect the changes in GBM-related miRNA expression profile. Twelve tumor-related miRNAs were identified in tumor tissues and compared with exosomal miRNA in patients with GBM. The expression of five miRNAs in serum exosomes was highly consistent with that in tumor tissues, which further confirmed the diagnostic value of circulating exosomal miRNAs in gliomas.

It has been well accepted that miR-21 is the earliest biomarker related to the diagnosis of glioma. Shi et al. (48) compared the levels of exosomal miR-21 in cerebrospinal fluid samples of glioma patients and non-tumor patients with brain trauma, and the results showed that the expression of exosomal miR-21 in cerebrospinal fluid of glioma patients was significantly higher than normal control (p < 0.001) and could be used as a biomarker for glioma diagnosis [area under the curve (AUC) = 0.872]. Santangelo et al. (99) collected blood samples from patients with different grades of gliomas before and after surgery and discovered that the expression levels of miR-21, miR-222, and miR-124-3p in the high-grade glioma group were significantly higher compared to those in the low-grade glioma group and the healthy control group (p < 0.001), and the expression levels of miR-21, miR-222, and miR-124-3p in serum were significantly decreased after operation (p <0.05). The expression level of miR-21, miR-222, and miR-124-3p in serum may serve as a valuable marker for the diagnosing and grading malignant gliomas (AUC for miR-21, miR-22, and miR-124-3p is 0.84, 0.80, and 0.78, respectively).

Meanwhile, Yang et al. (93) proved that miR-221 expression in the exosome was related to the early diagnosis and the WHO grade of glioma. The expression of miR-221 in exosomes of high-grade gliomas was higher than that of normal controls, which further confirmed the potential of exosomal miR-221 as a biomarker for diagnosis value of glioma (AUC = 0.78). Some studies have shown that the expression of serum exosomal miR-301a in patients with GBM was significantly higher than that in healthy controls (p < 0.01) and also associated with early diagnosis (AUC = 0.83). The level of serum exosomal miR-301a decreased significantly after surgical resection of the primary tumor and increased in tumor recurrence (p < 0.05) (104). The serum exosomal miR-301a may reflect the disease progression and pathological changes in patients with glioma.

To sum up, more and more studies have confirmed that there is a definite correlation between exosomal miRNAs and the specific diagnosis and pathological grading of gliomas, coupled with the fact that exosomes widely and stably exist in human body fluids (blood, cerebrospinal fluid, semen, urine, etc.). In the future, the gene detection of exosomal miRNAs may provide a better choice for early diagnosis and pathological grading of gliomas.

### Prognosis

Exosomal miRNAs can be used as biomarkers for the diagnosis of glioma and also as biomarkers for the prognosis evaluation of glioma. Zhuo et al. (105) conducted a meta-analysis and discovered that the poor prognosis was associated with the upregulation of 22 exosomal miRNAs in all solid tumor, including glioma. Shi et al. (106) measured the expression of miR-21 in tissue, cerebrospinal fluid, and plasma exosomes and found that the overall survival time of patients was negatively correlated with the expression level of exosomal miR-21 (p < 0.001). At the same time, Shi’s team compared the anatomical location, recurrence time, and recurrence interference of recurrent gliomas and discovered a significant correlation between the expression level of exosomal miR-21 in cerebrospinal fluid and the anatomic location of spinal/ventricular metastasis and recurrence of the tumor (p < 0.05). In another study, Lan et al. (104) found that the expression level of serum exosomal miR-301a in glioma patients was significantly higher than that in healthy controls, and the increase in exosomal miR-301a expression was associated with the progression of pathological grade and the decrease of Karnofsky functional status score (KPS). More importantly, they also found that the expression of serum exosomal miR-301a decreased significantly after surgical resection of the primary tumor, while it increased during recurrence. In the subsequent evaluation of the Kaplan–Meier survival curve, the overall survival rate of patients with low expression of plasma exosomal miR-301a was significantly higher compared to those with high expression levels [hazard ratio (HR) = 2.31, p < 0.001]. Microvessel density (MVD), as an important prognostic indicator of GBM, was found to be a better prognostic index than tumor type, stage, and grade in a gastric cancer study (107). Wang et al. (108) found that the overexpression of exosomal miR-26a derived from glioma stem cells promoted the proliferation and angiogenesis of human brain microvascular endothelial cells *in vitro* by inhibiting protein tyrosine phosphatase (PTP) gene which can also be observed *in vivo* experiments. Meanwhile, the higher expression of exosomal miR-26a related with short overall survival (OS) in glioma patient (HR = 1.42, p < 0.05). Vander et al. (101) and others found that miR-10b was highly expressed in tumor cells in central nervous system but not in normal brain tissues and was positively correlated with the malignant degree of the tumor. A high concentration of circulating exosomal miR-10b was also detected in cerebrospinal fluid of high-grade patients, And related to OS (HR = 1.36, p = 0.001). Thus, miR-10b may be used as a specific tumor marker to predict the prognosis.

Therefore, in view of the unique biological characteristics of exosomes, the collection of exosomes from patients’ body fluids, the detection of related miRNAs, combined with relevant bioinformatics analysis and processing, have a bright application prospect in the evaluation of treatment effect, survival time and even predicting the location of recurrent tumor in glioma patients

## Stem Cells-Derived Exosomal miRNAs in Glioma: Opportunities and Challenges

One obstacle that needs to be overcome in glioma treatment development is the existence of the blood–brain barrier. Most glioma chemotherapeutic drugs have low solubility in the blood, short half-life, and poor drug utilization rate. The efficacy of conventional drug administration is not satisfied (109). Currently, many attempts have been developed to efficiently and selectively deliver drugs to tumor sites by encapsulating the drugs in tumor-targeting carriers or nano-carriers which already achieved preliminary satisfactory results. However, the artificial properties of nano-carrier systems lead to toxicity-related problems *in vivo*. Therefore, there is an urgent need to build a non-toxic and efficient delivery carrier.

As a natural carrier of miRNAs, exosome is a nanoscale membranous vesicle with good circulatory stability, biological permeability, low immunogenicity, and toxicity. It can pass through the blood–brain barrier and blood–cerebrospinal fluid barrier without triggering immune rejection. In recent years, with the in-depth study of exosomal miRNAs molecular regulatory network, it has been confirmed that exosome transporting specific miRNAs can regulate and change the biological characteristics of glioma cells, therefore effectively inhibiting the malignant development of glioma (110).

Bone marrow mesenchymal stem cells (BMSCs) have been used for the packaging, production, and treatment in the field of anti-glioma miRNAs. Lang et al. (55) used miR-124a lentiviral vector to transfect bone marrow mesenchymal stem cells and isolated exosomes from the medium supernatant. After coculture, they discovered that the survival rate and proliferation of glioma stem cells decreased significantly. Furthermore, mice with intracranial glioma stem cell transplantation treated with isolated bone marrow mesenchymal cell exosomal miR-124a were associated with prolonged survival time. This process indicated that the exosomes can selectively carry miR-124a as a carrier to inhibit glioma growth and invasion. The main mechanism is that miR-124a overexpression decreases the level of target fork head box protein A2 (FOXA2) and leads to lipid accumulation in cells, so that glioma stem cells cannot effectively metabolize lipids, resulting in poisoning. Kim et al. (57) also indicated that the overexpression of exosomal miR-584 in U87 cells showed an increasing apoptosis rate and impaired proliferation and migration. Further animal studies *in vivo* showed that U87 tumor transplanted mice exposed to exosomes overexpression of miRNA-584 showed suppressed growth. Above studies suggested that exosomes secreted by BMSCs can be used as an anticancer vector to regulate tumor progression.

Recently, a new cell-free therapy was proposed to modify exosomes to inhibit glioma progress. Monfared team (111) constructed an “engineering exosome” that packaged miR-21-sponge and discovered that it could decrease the proliferation rate and increase the apoptosis rates of glioma cell lines U87MG and C6. To confirm the results, they also verified the tumor volume in the glioma rat model. The result showed that modified exosome significantly decreased the tumor volume compared to controls. Besides, Fareh et al. (59) tried to develop a new cell-free therapy by using glioma cells to secrete exosomal miRNAs. They combined glioma stem cells secreted miR-302-367 exosomes and human-derived glioma stem cells into the brains of immunodeficient mice and discovered that glioma stem cells overexpressing miR-302-367 could inhibit the proliferation of adjacent tumor cells *in vivo*. This strategy is particularly effective because it affects tumorigenesis and has a long-term inhibitory effect on tumors that have already been formed. In general, selective knockout or inhibition of genes related to tumor progression, or overexpression of related key tumor suppressor genes, will bring new hope to the field of glioma targeted therapy.

## Conclusions and Future Perspectives

Exosomes will change and supplement the traditional understanding of tumorigenesis and development and contribute to a comprehensive understanding of tumor-related molecular mechanisms Exosomes represent a new way of cell communication, which mediates cell communication between parent cells and target cells, and the transformation of the tumor microenvironment, in which both contribute to the malignant progress. Because of its specific advantages, they have excellent potential to be used in the diagnosis and detection of gliomas. However, the isolation, purification, and qualitative analysis of exosomes need to be further improved and optimized. Efficient and specific biomarkers also need to be further excavated. In addition, the precise treatment of tumors based on exosomes further enhances its effectiveness and drug utilization. These characteristics make exocrine-based precision therapy a new strategy for the treatment of gliomas. However, when using exosomes for the treatment of glioma, it is necessary to further clarify the mechanism of action of specific active components in exosomes and the role of glioma and improve its specificity. Standardizing the dreg dosage is also a problem that needs to be solved.

The types and levels of miRNAs in exosomes in the circulatory system are more closely related to glioma compared to free miRNAs. Other than free miRNAs, their level changes may be related to the WHO grade of glioma. Thus, they can provide a more sensitive and specific diagnostic method for glioma. However, there is evidence that no differential expression of exosomal miRNAs and free miRNAs in the circulation was detected between patients with glioma and the controls. Therefore, whether circulating exosomes miRNAs can be used as biomarkers for the diagnosis and prognosis of glioma is still questionable. We need studies with larger number of patients and the kinds of miRNAs to determine whether there are significant changes in the levels of circulating exosomal miRNA in glioma, whether there is a correlation between these changes and the pathophysiological changes in glioma, and whether the sensitivity and specificity of these new diagnostic methods are better than those currently used in clinical practice.

Glioma may affect circulating exosomal miRNAs, and exosomes derived from stem cells can also play an important role in glioma progress. They may serve as a new strategy for treating or preventing glioma in the future. However, There are still several hurdles before achieving clinical application, and this is a new beginning. First of all, the malignant progression of glioma is a complex biological process involves numerous factors and signal pathways. Any single mechanism cannot fully explain the changes in its biological characteristics. At present, most studies on glioma exosomal miRNAs are limited to the differential changes in expression profile, and research on how to regulate the mechanism of target genes and related signal pathways in receptor cells is still relatively limited. Second, as the specific sorting mechanism of exosome miRNAs and their immune regulation will cause adverse reactions or even toxicity to the body, it may cause damage to normal nerve tissue or other normal cells due to the specific sorting mechanism of exosomal miRNAs and its immune regulation that can cause adverse reactions or even toxicity still unknown. Third, the mostly exosomal miRNAs for diagnosis and treatment of glioma is still in the stage of experiment cells *in vitro* and *in vivo* animal experiment. How to achieve human trials and transform them into clinical diagnosis and treatment remains a challenge. The mechanism of exosome miRNAs achieving their specificities are not fully understood. Therefore, the administration of exosomal miRNA cell-free therapy still encounter many challenges. We need to further analyze the roles of different miRNAs derived from glioma, astroglial cell, and stem cells exosomes and clarify their positive roles in the pathophysiological process of glioma and the fatal effects of exosomal miRNAs on normal tissues. With the further research on exosomes and exosomal miRNAs, the above problems will be solved one by one, which will provide a solid and reliable theoretical basis for the diagnosis and treatment of glioma, and eventually bring about remarkable progress.

## Author Contributions

AY drafted the manuscript. MY and MN retrieved the relevant literature. HQ made a statistical table. ZW and YW designed the structure of this article and reviewed the final manuscript prior to submission. All authors contributed to the article and approved the submitted version.

## Conflict of Interest

The authors declare that the research was conducted in the absence of any commercial or financial relationships that could be construed as a potential conflict of interest.

## Publisher’s Note

All claims expressed in this article are solely those of the authors and do not necessarily represent those of their affiliated organizations, or those of the publisher, the editors and the reviewers. Any product that may be evaluated in this article, or claim that may be made by its manufacturer, is not guaranteed or endorsed by the publisher.

## Abbreviations

GBM, glioblastoma; CSF, cerebrospinal fluid; MVE, multivesicular endosome; HMVEC, human microvascular epithelial cell; FGF, fibroblast growth factor; VEGF, vascular endothelial growth factor; EGFRv III, epidermal growth factor receptor variant III; NTs, neurotrophic factor; ESCRT, endosomal sorting complexes required for transport; BBB, blood–brain barrier; P-gp, P-glycoprotein; MSC, marrow stromal cells; FOXA2, forkhead box protein A2; B-Exos, blood-derived exosomes; TMZ, temozolomide.
